# Auricular Acupressure Effect on Autonomic Responses Evoked by a Cold Pressor Test in Healthy Volunteers: A Pilot Study

**DOI:** 10.1155/2022/5703760

**Published:** 2022-12-13

**Authors:** Dieu-Thuong Thi Trinh, Van-Huy Nguyen, Minh-Man Pham Bui, Van-Dan Nguyen, Thu-Thao Thi Nguyen, Hoai-Nam Thai, Khac-Minh Thai

**Affiliations:** ^1^Faculty of Traditional Medicine, University of Medicine and Pharmacy at Ho Chi Minh City, Ho Chi Minh, Vietnam; ^2^Grant and Innovation Center, University of Medicine and Pharmacy at Ho Chi Minh City, Ho Chi Minh, Vietnam; ^3^University Medical Center Ho Chi Minh City, University of Medicine and Pharmacy at Ho Chi Minh City, Ho Chi Minh, Vietnam; ^4^Faculty of Pharmacy, University of Medicine and Pharmacy at Ho Chi Minh City, Ho Chi Minh, Vietnam

## Abstract

**Objective:**

This pilot study was conducted to investigate changes in the pulse rate and blood pressure in healthy volunteers after applying auricular acupressure at the “heart acupoint.”

**Methods:**

A total of 120 healthy volunteers with hemodynamic indexes within normal limits were randomly allocated into 4 groups to receive auricular acupressure treatment either at the heart acupoint of the left or the right, or in both ears, and one control group without applying auricular acupressure.

**Results:**

Before the application of auricular acupressure, there were no statistical differences in pulse rate and blood pressure increments among the four groups during the first cold pressor test. In groups in which auricular pressure was applied, the pulse rate was significantly reduced after the application of auricular acupressure in three groups; however, no statistically significant difference was detected among the groups. Changes in blood pressure were not statistically significant in or among the different groups after applying auricular acupressure. The average recorded pulse rate values during the second cold pressor test (after auricular acupressure) were significantly lower compared to the corresponding values taken during the first cold pressor test (before auricular acupressure) (*p* < 0.05); however, pulse rate increments during the two cold pressor tests (with and without auricular acupressure) were similar (*p* > 0.05).

**Conclusions:**

These findings suggest that auricular acupressure could be used as an adjunctive nonpharmacological method for reducing the pulse rate.

## 1. Introduction

Inappropriate sinus tachycardia (IST) is a clinical entity characterized by an unexplainable resting heart rate greater than 100 beats per minute (bpm) with an average heart rate exceeding 90 bpm on a 24-hour Holter monitor, in symptomatic patients experiencing palpitations, dyspnea, or dizziness in the absence of any primary cause of tachycardia [1–3]. The prevalence of IST is estimated to be approximately 1.16% [4]. It is suggested that the general mechanisms behind the occurrence of IST could be either an inherent increase in the sinus node automaticity or an extrinsic imbalance between sympathetic and parasympathetic (vagal) influence over the heart rate. Another evidence suggested a potential relationship between IgG anti-*β* receptor antibodies and IST by potentiating extended calcium influx and depolarization within *β*-adrenergic receptors leading to prolonged activation and an increase in heart rate [5]. More recent evidence points to a possible genetic class of IST that was linked to a gain-of-function mutation in the HCN4 pacemaker channel causing increased sensitivity to the second messenger cAMP, leading to an increase in sympathetic tone [6].

Several reports have described the role of auricular acupuncture, which uses needles to stimulate acupuncture points (acupoints) in specific zones of the ear, in inducing vagal tone as a counteractive measure to ameliorate patients' physical, emotional, and neurological symptoms and signs [7–9]. It was largely believed that stimulating certain zones within one's ear, specifically zone CO15 (“heart acupoint”), influences the stimulation of the vagus nerve with a subsequent decrease in heart rate, regulation of blood pressure, and improvement of chest pain [10, 11].

Auricular acupressure (AA) is a noninvasive form of auricular acupuncture, which uses physical pressure applied to acupoints by Vaccaria seeds [12]. There is limited evidence about AA's efficacy, particularly in volunteers with an increased sympathetic tone. One report demonstrated the utility of AA in reducing the side effects of chemotherapy in women with ovarian cancer [13]. A randomized controlled trial on postpartum women found that women who received AA had significantly lower average cortisol levels, heart rate, anxiety, and fatigue than women who were in the control group 5 days after delivery [14]. Similarly, another study found significant differences in pain control and mental health in hypertensive patients undergoing AA versus those who did not, despite not being able to find significant differences in other parameters like heart rate and quality of life in such patients [15]. On the other hand, the effect of stimulation at vagus branches either at the left, right, or both ears in hemodynamic indices is still vague and different between experimental models and humans [16].

The cold pressor test (CPT) was developed as a safe method to induce experimental sinus tachycardia and blood pressure acceleration by increasing the sympathetic tone [17].

By using CPT, we aimed to make the volunteer's heart rate (also known as pulse rate) and blood pressure increased to examine the AA's effectiveness. This study was designed to investigate changes in pulse rate and blood pressure in healthy volunteers after applying AA either at the “heart acupoint” of the left or the right, or in both ears while using CPT. Furthermore, we aimed to compare the resulting changes in pulse rate and blood pressure across three groups as well as investigating possible side effects of both CPT and AA.

## 2. Methods

### 2.1. Study time

The study time was from December, 2017, to June, 2018.

### 2.2. Design

This study was designed as a pilot study in which 120 healthy volunteers were recruited by the research team at the Faculty of Traditional Medicine, University of Medicine and Pharmacy in Ho Chi Minh City, Vietnam. The study protocol and Institutional Review Board (IRB) documents were submitted to the ethical committees in the designated medical center for approval.

The sample size was calculated according to the formula *n*=2*xCx*(1 − *r*)/*ES*^2^.

#### 2.2.1. Formula Note

*n* is the number of sample sizes needed for the study; *r* is the correlation coefficient between the two measurements. ES is the influence coefficient estimated by the formula: ES = *d*/*s*. *d* and *s* are the average and standard deviation of pulse rate when conducting CPT, respectively. According to Saab et al., the mean standard deviation of heart rate when doing CPT in the feet is 13.2 beats/minute [18]. With the expectation that AA can help reduce pulse rate by 5 beats/minute during CPT, we calculated *n* = 29.30. So, the sample size for each group is 30 and the total sample of the study is 120.

### 2.3. Participants

Following the signing of written informed consent, participants were randomly allocated into 4 groups (30 people in each group) using GraphPad software to receive AA treatment either at the heart acupoint of the left (1), the right (2), both ears (3), and one control group without AA application (0). They were then monitored to determine the efficacy of the given treatment.

Participants were individuals aged between 18 and 30 years, with a body mass index (BMI) of 18 to 23 kg/m^2^, pulse rate ranging between 60 and 99 beats per minute (bpm), supine arterial pressures of less than 140/90 mmHg, not suffering from any medical conditions and willing to consent to the study terms and protocol. They were also committed to come on the study day devoid of drugs, smoking, and caffeine as well as stress (as evidenced by a Depression Anxiety Stress Scale-21 stress point measure of less than 15 points). Exclusion criteria included volunteers who were outside of the age range, suffered from acute diseases, using stimulants (coffee, alcohol, and tobacco) within 24 hours before the study, exercising immediately before the study, or taking medications affecting pulse rate and blood pressure within the previous month. Also, pregnant or menstruating females were excluded.

### 2.4. Auricular Acupressure

We selected heart acupoints (CO15), located in the middle of the ear cavity, as suggested in the nomenclature and location of auricular points [19]. Irrespective of laterality, ears were cleaned with 70% alcohol both before and after applying the acupressure ear seeds. The acupuncturist attached an adhesive plaster of dimensions 1 × 1 cm with one bead (Semen Vaccariae, Taicheng Technology and Development Co. Ltd., Shanghai, China). The globes were smooth-surfaced, black in color, and approximately 2 mm in diameter. Every 5 minutes, the acupuncturist manually stimulated the heart acupoint by pressing down with a gentle force until the sensation of burning, soreness, numbness, distension, or heat is felt (such a sensation is called “de qi”) for approximately 30 seconds. [20] Seeds were left at the heart acupoints while volunteers were resting for 17 minutes and were then removed. The acupuncturist was trained at the University of Medicine and Pharmacy of Ho Chi Minh City and had 5-year experience in performing AA.

### 2.5. Cold Pressor Test

Participants had to immerse their feet in cold water (7°C) for 3 minutes under supervision [21]. The water was initially poured into a 20 × 50 cm cylinder tub. Then, ice was added and the water was stirred until a temperature of 7°C was reached. After CPT, participants were given a towel to dry their feet and take rest in the supine position.

### 2.6. General Protocol

**Figure 1:**
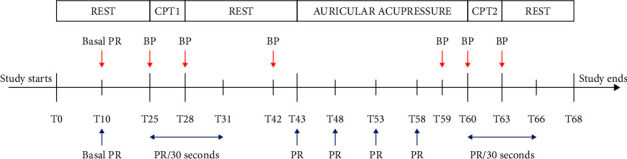
Study protocol. The study protocol includes 6 stages: rest, first cold pressor test, rest, auricular acupressure, second cold pressor test, and rest. BP: measure blood pressure, PR: measure pulse rate, PR/30 seconds: measure pulse rate every 30 seconds, CPT1: first cold pressor test, CPT2: second cold pressor test.

The study was carried out in a quiet room with an air temperature of 26°C. The volunteers lied comfortably in a semi-recumbent position. After a 10-minute rest, baseline values of the pulse rate (PR) and blood pressure (BP) (systolic and diastolic) were taken (T10). At T25, the first CPT (CPT1) was started. PR was recorded continuously over 6 minutes from T25 to T31 (3 minutes during CPT and 3 minutes after CPT has ended) using an oximeter (GIMA OXY-200, Italy) in the right hand. Simultaneously, BP was measured twice at the beginning and at the end of CPT (T25 and T28), using a digital blood pressure monitor (OMRON HEM 7121, Japan) in the left hand. After the second period of rest (between T31 and T43), AA was started at T43 and lasted for 17 minutes (till T60). The practitioner stimulated the heart acupoint every 5 minutes (at T43, T48, T53, and T58). PR was measured before and after each stimulation, and BP was measured before the beginning of AA (at T42) and at its end (at T59). Then, the second CPT (CPT2) started, and the PR was continuously recorded over 6 minutes from T60 to T66 (3 minutes during the CPT and 3 minutes after its end). BP was measured twice; at the beginning and at the end (T60 and T63, respectively). The study protocol is shown in Figure 1.

### 2.7. Analysis Strategy

The analysis of the data was performed by using STATA 13 software. A dependent *t*-test or ANOVA test was used to compare changes in age, PR, and systolic and diastolic BPs within and among four designated groups at certain time points (and a post-hoc test was also used if needed), and a chi-squared test was used to check the compare the gender variables among the four groups. Additionally, a mixed-effects model for time series was used to compare PR increments between two intervals of CPT. For all the above statistical tests, a *p* value of less than 0.05 was considered statistically significant. All data were transferred to the blinded analyst.

### 2.8. Ethical Considerations

There was no patient involved. Ethical approval for this study was obtained from the Board of Ethics in Biomedical Research of the University of Medicine and Pharmacy in Ho Chi Minh city.

## 3. Results

There were no participants who discontinued or were excluded from the trial. It took 68 consecutive minutes to finish the trial for one person. Data collection time was 50 days.

### 3.1. General Characteristics of the Study Population

Table 1 shows the general characteristics of the study volunteers included in each group at the start of the experimental protocol. It is shown that they were age-matched (*p* > 0.05, ANOVA test) and the DBP was significantly different across the four designated study groups (*p* < 0.05, ANOVA test). More specifically, the basal DBP in group 0 was significantly higher when compared to group 1 (*p* < 0.05, *t*-test).

### 3.2. Pulse Rate during the Experiment

#### 3.2.1. Pulse Rate during the First Cold Pressor Test

**Figure 2:**
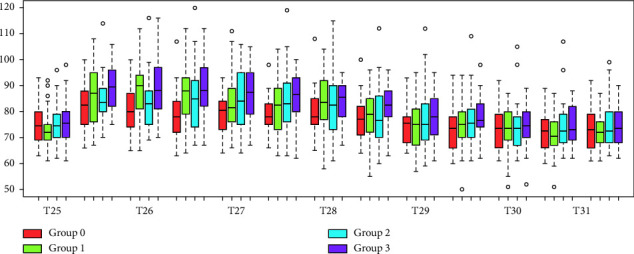
Pulse rate during the first cold pressor test. The figure demonstrates the pulse rate of the four study groups during the first cold pressor test (from T25 to T28) and 3 minutes after the first cold pressor test (from T28 to T31).

During CPT1, it was observed that the difference in PR at T10 (basal PR) and T25 (starting CPT1) was not statistically significant among groups (*p* > 0.05, *t*-test), except for group 0 where PR at T25 was higher compared to basal PR.  Figure 2 shows that during the CPT1, statistically significant differences in the recorded average PR from that recorded at the start of the test within each participating group were observed during the first seven 30-second intervals (*p* < 0.05, *t*-test). Over the consecutive time intervals after the 210th second, changes in the recorded average PR from baseline values were not statistically significant (*p* > 0.05, *t*-test). It was also noticed that from T25 to T31, differences in the average PR values recorded for each time interval relative to baseline were not statistically significant among the 4 participating study groups (*p* > 0.05, ANOVA test). Based on the results of the mixed-effects model, PR increments among the 4 groups were similar (*p* > 0.05, mixed-effects model). After the end of the CPT1, the PR of groups 2 and 0 returned to normal at T28.5 (30 seconds later) (*p* > 0.05, *t*-test), meanwhile PR of groups 1 and 3 became normal at T29 (60 seconds later).

#### 3.2.2. Pulse Rate during the Auricular Acupressure Stage

**Figure 3:**
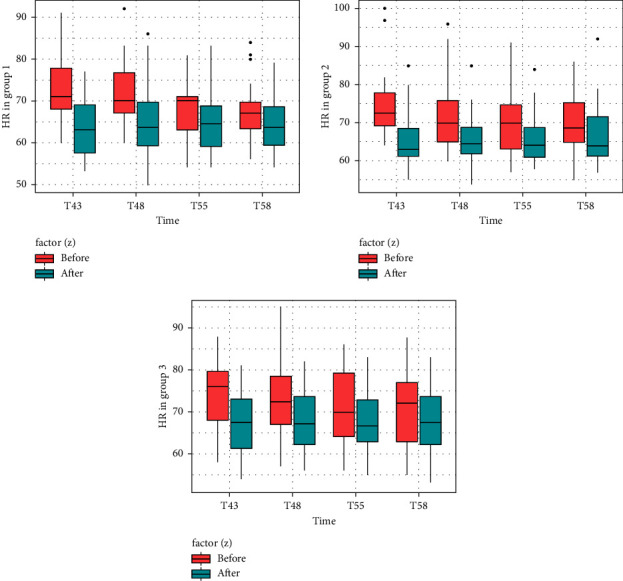
Pulse rate during the auricular acupressure stage. The figure demonstrates the pulse rate in the three groups which auricular acupressure was applied before and after acupoint stimulation (at T43, T48, T53 and T58). The red rectangle and the blue rectangle are the pulse rates before and after acupoint stimulation, respectively.

Table 2 shows that in groups 1, 2, and 3, PR before the auricular acupressure stage (T43a) was not statistically different from basal PR (T10) (*p* > 0.05, *t*-test).  Figure 3 shows that PR changes before and after manipulating the heart acupoint (at T43, T48, T53, and T58, with 30 seconds/time). It is evident that the PR decreased after every manipulation. It is shown in Table 2 that for all involved study groups, the median PR measurements recorded at the end of the acupressure stage (T60) were significantly less than those recorded at the start of the acupressure stage (T43a) but within the acceptable boundaries of a normal PR (*p* < 0.05, *t*-test). In detail, in group 1, PR at T60 (65, 62–72) was lower than T43a (71, 67–78) (*p* < 0.001, *t*-test). Similarly, in group 2, PR at T60 (71, 63–77) was lower than T43a (72.5, 69–78) (*p* = 0.001, *t*-test), and in group 3, PR at T60 (70.5, 63–77) was lower than that at T43a (76, 68–80) (*p* = 0.007, *t*-test). Also, when comparing PR before each manipulation to the basal PR (T10), except for group 1, the PR was found to be significantly less than that before the second manipulation (*p* < 0.05, *t*-test).

### 3.3. Comparison of Pulse Rate between Two Cold Pressor Tests

**Figure 4:**
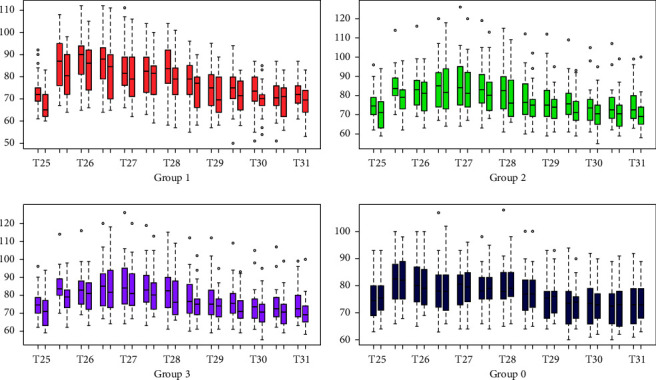
Pulse rate during the first and second cold pressor test. The figure demonstrates the comparisons of the pulse rates at the corresponding times between two cold pressor tests, the front rectangle is pulse rate at the first cold pressor test, and the back one is the pulse rate at the second cold pressor test. There are a total of 13 comparison times.

Figure 4 demonstrates the differences among groups about the average recorded PR values that were taken during the CPT2 and compared to their corresponding values that were previously measured across the same time points during the CPT1. It was evident that within each study group and across all time points from T60 to T66-the average recorded PR values during the CPT2 were significantly lower as compared to the corresponding values taken during the CPT1 (*p* < 0.05, *t*-test), except for group 0 (*p* > 0.05, *t*-test). As for the in-between group differences (groups 1, 2, and 3) in the recorded average PR values across every time point from T60 to T66, results showed that those differences were not statistically significant (*p* > 0.05, ANOVA test). Based on the results of the mixed-effects model, PR increments among the 4 groups were similar (*p* > 0.05, mixed-effects model).

### 3.4. Blood Pressure during the Experiment

#### 3.4.1. Blood Pressure during the First Cold Pressor Test

Tables 3 and 4 show systolic blood pressure (SBP) and diastolic blood pressure (DBP) at different experimental stages. SBP and DBP increased to peak at T25 and gradually decreased at T28 but were still higher than basal SBP and DBP (measured at T10) (*p* < 0.05, *t*-test), and then returned to normal at T42 before the AA stage beginning (*p* > 0.05, *t*-test). However, when comparing changes in SBP and DBP among groups at corresponding time intervals, the results were not statistically significant (*p* > 0.05, ANOVA test).

#### 3.4.2. Blood Pressure during the Auricular Acupressure Stage

When comparing SBP and DBP measured at the time before (T42) and after (T59) the AA stage in each group as well as comparing differences among groups, the results were not statistically significant (*p* > 0.05, *t*-test, and ANOVA test).

### 3.5. Comparison of Blood Pressure between Two Cold Pressor Tests

When compared, the recorded average SBP and DBP between CPT1 (T25–T28) and CPT2 (T60–T63) within each group as well as among groups showed no statistically significant differences (*p* > 0.05, *t*-test, and ANOVA test).

## 4. Discussion

Aiming to explore an everlasting knowledge gap concerning the effect of auricular stimulation of the heart acupoint on the autonomic input to the cardiovascular system, we conducted this study using an experimental protocol to validate whether right, left, or bilateral stimulation of the heart acupoint would cause a significant difference in cardiovascular outcomes like PR, SBP, and DBP among the recruited study groups. Immersion of participants' feet in cold water during the cold pressor test was conducted as it was proven to be associated with cardiovascular changes with significant pulse rate and blood pressure acceleration [17].

In comparing PR and BP changes before and after acupressure, it was noted that although SBP and DBP did not differ significantly before and after AA, every manipulation to the heart acupoint significantly reduced the post-manipulation PR in comparison to its pre-manipulation value. Moreover, the end-point PR was significantly lower than the starting PR at the beginning of the AA stage. Results pertinent to a decrease in PR were similar to those obtained by Gao et al. who investigated the effect of manual and vibratory ear stimulation on heart rate as well as other parameters in a cohort of 14 healthy volunteers of comparable anthropometric characteristics [10]. In contrast, a recently published systematic review and meta-analysis investigating the acute effects of transcutaneous electrical nerve stimulation (TENS) on heart rate, systolic blood pressure, and diastolic blood pressure in groups of healthy adults above 18 years of age revealed a statistically significant reduction in heart rate (−2.55 bpm; 95% CI = −4.31 to −0.78; *I* (2) = 86%; *p* = 0.005), systolic blood pressure (−3.00 mmHg; 95% CI = −5.02 to −0.98; *p* = 0.004), and diastolic blood pressure (−1.04 mmHg; 95% CI = −2.77 to −0.03; *I* (2) = 61%; *p* = 0.04) using TENS of various frequencies [22]. Moreover, an earlier animal study was conducted using different auricular areas and different methods of auricular stimulation in mice found that the greatest reduction in heart rate and blood pressure came from stimulating the inferior concha (−18.4 bpmf ± 3.1 mmHg, *p* < 0.001) with milder reductions occurring after using other areas and using the manual method of auricular stimulation as compared to the electroacupuncture [7]. The external ear is the only place on the body where the vagus nerve sends its only peripheral branch. In fact, the auricular branch of VN surfaces as the afferent auricular vagus nerve and thus forms a cutaneous receptive field in the pinna of the ear. This field is susceptible to external stimuli in terms of peripheral nerve stimulation. The afferent vagus nerve is composed out of myelinated A*β* fibers (fibers with a diameter of 7–10 *μ*m comprise about 20% of the total myelinated aVN axons, with about 370 myelinated axons per auricle), myelinated A*δ* fibers (fibers with the diameter of 2–5 *μ*m comprise about 50% of the total myelinated aVN axons), and non-myelinated C fibers. The recruitment of different fiber types depends on the threshold stimulus that is determined by the total charge delivered to the nerve fiber [16, 23]. The observation in previous studies suggests that needle insertion, i.e., manual needle manipulation and strong electroacupuncture (EA), which stimulate needles by electricity, are effective in evoking autonomic responses, whereas weaker forms of EA are not. Gao et al. suggest that strong or even painful stimulation may be required to produce these responses. This may be because EA stimulates local receptors as well as nerve fibers that innervate other areas passing through the area of stimulation and that the effective stimulation area of electroacupuncture may well exceed the area between the two inserted needles as a result of the spreading of the electrical current. In contrast, manual manipulation stimulates receptors primarily at the site of needle insertion [7]. Therefore, acupressure, which is a noninvasive method and does not use electricity, could not create a strong stimulation enough to the vagus nerve branches to significantly affect both pulse rate and blood pressure. More studies with larger sample sizes should be conducted to verify that claim.

With respect to comparing changes in PR and BP among the participating study groups (groups 1, 2, and 3), we found no significant differences in the abovementioned cardiovascular outcomes, implying that there was no difference in the desired outcomes no matter what the site of auricular stimulation was (right, left, or both heart acupoints). Unfortunately, that was not the case in previous literature comparing the above outcomes according to the laterality of stimulation in the animal as well as human models [16, 24]. Strauss and colleagues, in congruence with anatomical conventional wisdom, were able to prove that, in rats, left‐sided cervical vagal nerve stimulation (VNS) potentiated stronger bradycardic and hypotensive effects than right‐sided VNS [16]. In contrast, other studies have shown that a much greater depression in heart rate and blood pressure occurred with the right VNS as compared to the left [25, 26]. Chen et al. argued that that controversy was anticipated as there was no evidence to prove that stimulation of either side of the auricular branch of the vagus nerve (ABVN) produced more depression in cardiovascular outcomes than the other side [24]. They went on to explain that by drawing on the neurocircuitry of ABVN stimulation. According to Chen et al., stimulation of either tragus activates the corresponding ABVN, whose afferent fibers deliver stimulatory signals to the nucleus tractus solitaries (NTS), which in turn, integrates these stimuli and then projects these processed signals to the heart through either branch of the right or left vagus nerves, leading to an equally created outcome irrespective of the initial side of tragus stimulation [24].

Although the PR and BP increments of the two times CPT were similar, the PR values during the CPT2 (after auricular acupressure) were significantly lower as compared to the corresponding values taken during the CPT1 (before auricular acupressure), which has an important role in preventing excessive hemodynamic parameters when encountering acute stress. This phenomenon can be explained in terms of the fact that parasympathetic stimulation overrides the sympathetic one, a fact that has been proven to be extremely beneficial in cardiac failure patients due to their autonomic imbalance that would increase their risk of mortality [22, 27]. Side effects such as pain (measured by the Visual Analogue Scales) and syncope (due to vasovagal reactions) were not encountered during our experiment, either during the AA or CPT tests. Such findings were equally reported by previous studies analyzing the safety of auricular stimulation procedures, suggesting the relative safety of the use of this test for future studies [28, 29].

This is one of the first trials in comparing effects on hemodynamic indexes of auricular acupressure at heart acupoint of the left, right, and both ears. Our research has a number of limitations: we only recorded the PR every 30 seconds, so we could not find exactly when the PR returned to normal. SBP and DBP were only measured two times (at the beginning and end of the CPT). Future studies are suggested to continuously monitor these parameters.

## 5. Conclusion

In conclusion, using the pilot study design, auricular acupressure did not affect PR increment in the CPT2, but it reduced PR values during the CPT2 (after auricular acupressure) when compared to the corresponding values taken during the CPT1 (before auricular acupressure). In groups in which AA was applied, every time the heart acupoint was manipulated, the PR was significantly reduced. Besides, auricular acupressure did not affect BP and there were no side effects were recorded when conducting auricular acupressure as well as CPT. Our study is among the pioneering research in comparing effects on hemodynamic indexes of auricular acupressure at the heart acupoint of the left, right, and both ears.

## Figures and Tables

**Table 1:** General characteristics of the study sample (*N* = 120).

Characteristics	Group 1 (left ear) (*N* = 30)	Group 2 (right ear) (*N* = 30)	Group 3 (both ears) (*N* = 30)	Group 0 (control group) (*N* = 30)	*p* value
*Gender (No., %)*					
Male	15 (50%)	14 (47%)	14 (47%)	14 (47%)	0.9920
Female	15 (50%)	16 (53%)	16 (53%)	16 (53%)	
Age (years)	23 (22–23)	22 (21–24)	23 (22–23)	23 (22–23)	0.8422
Median (IQR)					
PR (bpm)	72 (67–78)	76.5 (71–85)	78 (69–82)	72.5 (66–76.75)	0.7140
Median (IQR)					
SBP (mmHg)	107 (104–111)	109.5 (101–116)	106 (101–111)	110 (101.25–120)	0.5697
Median (IQR)					
DBP (mmHg)	66 (63–68)	69 (63–71)	67 (61–69)	70 (65–70)	0.0409^*∗*^
Median (IQR)					

PR = pulse rate, SBP = systolic blood pressure, DBP = diastolic blood pressure, IQR = interquartile range. ^*∗*^significantly different. Statistical tests: Chi-squared test (Gender), ANOVA test (Age, PR. SBP, DBP).

**Table 2:** Pulse rate changes during the experiment.

Stage	Time	*Pulse rate (bpm) median (IQR)*				*Comparison to basal PR of each group p value*			
		Group 1	Group 2	Group 3	Group 0	Group 1	Group 2	Group 3	Group 0
Basal	T10	72 (67–78)	76.5 (71–85)	78 (69–82)	72.5 (66–76.75)				
CPT1	T25	72 (69–75)	74.5 (70–79)	75.5 (70–80)	74.5 (69.5––80)	0.3334	0.2004	0.7024	0.0015^*∗*^
	T28	83.5 (77–92)	82.5 (73–90)	85.5 (78–90)	78 (75.25–84.75)	<0.001^*∗*^	<0.001^*∗*^	<0.001^*∗*^	<0.001^*∗*^

Rest	T31	72 (68–76)	72.5 (68–80)	73.5 (68–80)	73 (66.50–78.25)	0.4974	0.0255^*∗*^	0.0430^*∗*^	0.4746
AA	T43a	71 (67–78)	72.5 (69–78)	72.5 (69–78)		0.7651	0.0588	0.0738	
	T43b	63 (57–69)	63 (61–69)	67.5 (61–74)		<0.001^*∗*^	<0.001^*∗*^	<0.001^*∗*^	
	T48a	70 (67–77)	70 (65–76)	72.5 (67–79)		0.0666	<0.001^*∗*^	0.0018^*∗*^	
	T48b	63.5 (59–70)	64.5 (62–69)	67 (62–74)		<0.001^*∗*^	<0.001^*∗*^	<0.001^*∗*^	
	T53a	70 (63–71)	70 (63–75)	70 (64–80)		<0.001^*∗*^	<0.001^*∗*^	0.001^*∗*^	
	T53b	64.5 (59–69)	64 (61–69)	66.5 (63–73)		<0.001^*∗*^	<0.001^*∗*^	<0.001^*∗*^	
	T58a	67 (63–70)	68.5 (65–76)	72 (63–77)		<0.001^*∗*^	<0.001^*∗*^	<0.001^*∗*^	
	T58b	63.5 (59–69)	64 (61–72)	67.5 (62–74)		<0.001^*∗*^	<0.001^*∗*^	<0.001^*∗*^	

CPT2	T60	65 (62–72)	71 (63–77)	70.5 (63–77)	75.5 (71.25–80)	<0.001^*∗*^	<0.001^*∗*^	<0.001^*∗*^	<0.001^*∗*^
	T63	79 (72–84)	76 (69–88)	81 (75–88)	79 (76.25–85)	0.0065^*∗*^	0.1207	0.0021^*∗*^	<0.001^*∗*^

Rest	T66	69.5 (64–74)	69 (65–74)	69.5 (63–74)	73 (68–78.75)	<0.001^*∗*^	<0.001^*∗*^	<0.001^*∗*^	0.1830

PR = pulse rate, IQR = interquartile range, AA = auricular acupressure, CPT1 = first cold pressor test, CPT2 = second cold pressor test, *a* = before acupoint stimulation, *b* = after acupoint stimulation. ^*∗*^significantly different. Statistical test: *t*-test.

**Table 3:** Systolic BP changes during the experiment.

Stage	Time	*Systolic blood pressure (mmHg) median (IQR)*				*Comparison to basal SBP of each group*			
		Group 1	Group 2	Group 3	Group 0	Group 1	Group 2	Group 3	Group 0
Basal	T10	107 (104–111)	109.5 (101–116)	106 (101–111)	110 (101.25–120)				
CPT1	T25	121 (113–130)	120.5 (110–128)	111 (121–130)	132 (122–135)	<0.001^*∗*^	<0.001^*∗*^	<0.001^*∗*^	<0.001^*∗*^
	T28	113 (108–119)	113.5 (106–119)	114.5 (105–124)	115 (110–123)	<0.001^*∗*^	0.0019^*∗*^	<0.001^*∗*^	<0.001^*∗*^

AA	T42	106.5 (104–109)	107 (101–113)	103 (97–107)		0.5948	0.0950	0.0780	
	T59	106.5 (102–109)	106.5 (99–113)	104 (99–110)		0.1883	0.0643	0.1040	

CPT2	T60	120 (115–127)	117.5 (109–125)	117 (112–126)	131 (123.5–34.75)	<0.001^*∗*^	<0.001^*∗*^	<0.001^*∗*^	<0.001^*∗*^
	T63	112 (106–118)	108.5 (102–115)	111 (105–118)	115 (110.25–122.75)	0.0033^*∗*^	0.7337	<0.001^*∗*^	0.0025^*∗*^

SBP = systolic blood pressure, IQR = interquartile range, AA = auricular acupressure, CPT1 = first cold pressor test, CPT2 = second cold pressor test. ^*∗*^significantly different. Statistical test: *t*-test.

**Table 4:** Diastolic BP changes during the experiment.

Stage	Time	*Diastolic blood pressure (mmHg) median (IQR)*				*Comparison to basal DBP of each group*			
		Group 1	Group 2	Group 3	Group 0	Group 1	Group 2	Group 3	Group 0
Basal	T10	66 (63–68)	69 (63–71)	67 (61–69)	70 (65–70)				
CPT1	T25	78.5 (71–85)	79 (76–82)	81 (75–88)	87.5 (82.25–91.75)	<0.001^*∗*^	<0.001^*∗*^	<0.001^*∗*^	<0.001^*∗*^
	T28	69 (65–63)	77.5 (66–78)	70 (64–77)	74.5 (67.25–79.5)	0.0024^*∗*^	<0.001^*∗*^	0.0229^*∗*^	<0.001^*∗*^

AA	T42	67 (65–69)	66 (64–71)	65.5 (63–69)		0.8584	0.6949	0.7713	
	T59	65 (63–69)	67 (65–72)	63.5 (61–69)		0.7583	0.5451	0.0563	

CPT2	T60	77.5 (72–82)	77.5 (72–82)	80 (75–86)	87.5 (84.25–89.75)	<0.001^*∗*^	<0.001^*∗*^	<0.001^*∗*^	<0.001^*∗*^
	T63	70 (64–75)	70 (65–76)	70 (65–74)	74.5 (69.25–78)	0.0172^*∗*^	0.0558	0.0536	<0.001^*∗*^

DBP = diastolic blood pressure, IQR = interquartile range, AA = auricular acupressure, CPT1 = first cold pressor test, CPT2 = second cold pressor test. ^*∗*^significantly different. Statistical test: *t*-test.

## Data Availability

The data used to support the findings of this study are available on request.
